# Validation of a multi-frequency bioelectrical impedance analysis device for the assessment of body composition in older adults with type 2 diabetes

**DOI:** 10.1038/s41387-022-00223-1

**Published:** 2022-10-20

**Authors:** Assaf Buch, Arie Ben-Yehuda, Vanessa Rouach, Andrea B. Maier, Yona Greenman, Elena Izkhakov, Naftali Stern, Roy Eldor

**Affiliations:** 1 Department of Nutritional Sciences, School of Health Sciences, Ariel University, Ariel, Israel; 2 Institute of Endocrinology, Metabolism, and Hypertension, Tel Aviv Sourasky Medical Center, Tel-Aviv, Israel; 3 Department of Medicine, Hadassah-Hebrew University Medical Center, Jerusalem, Israel; 4 Sackler Faculty of Medicine, Tel Aviv University, Tel-Aviv, Israel; 5 Department of Human Movement Sciences, @AgeAmsterdam, Amsterdam Movement Science, Vrije Universiteit Amsterdam, Amsterdam, The Netherlands; 6 Department of Medicine and Aged Care, @AgeMelbourne, The Royal Melbourne Hospital, The University of Melbourne, Melbourne, VIC Australia; 7 Healthy Longevity Translational Research Program, Yong Loo Lin School of Medicine, National University of Singapore, Singapore, Singapore; 8 The Sagol Center for Epigenetics of Metabolism and Aging, Tel Aviv Sourasky Medical Center, Tel-Aviv, Israel

**Keywords:** Body mass index, Geriatrics

## Abstract

**Background:**

Aging and type 2 diabetes (T2DM) are associated with an increased risk of sarcopenia. Diagnosis of sarcopenia is commonly done using dual-energy X-ray absorptiometry (DXA) in specialized settings. Another available method for assessing body composition is direct segmental multi-frequency bioelectrical impedance analysis (DSMF-BIA). Here, we examine the accuracy of a DSMF-BIA (InBody-770) for assessing body composition in older adults with T2DM when compared to DXA.

**Methods:**

Eighty-four obese/overweight older adults (49 women, 71 ± 5 years) with T2DM who were recruited for the CEV-65 study and had both DSMF-BIA and DXA assessments at baseline were included. The analysis included Bland–Altman plots and intra class correlation coefficients. Sub-analyses were performed according to gender and following 10 weeks of interventions (diet, circuit training, and Empagliflozin).

**Results:**

The leg lean mass results according to DSMF-BIA and DXA were 14.76 ± 3.62 kg and 15.19 ± 3.52 kg, respectively, with no difference between devices according to Bland–Altman analyses (*p* = 0.353). Assessment of appendicular skeletal mass index did not differ between DSMF-BIA and DXA (7.43 vs. 7.47 kg/m^2^; *p* = 0.84; ICC = 0.965, *p* < 0.0001; mean difference −0.068, *p* = 0.595). Gender and treatment interventions did not modify the accuracy of the DSMF-BIA when compared to DXA.

**Conclusions:**

In older adults with T2DM the degree of agreement between DSMF-BIA and DXA, was high, supporting the use of DSMF-BIA to measure muscle mass.

## Background

In western countries, the proportion of people over the age of 60 years is increasing faster than any other group [1]. Type 2 diabetes (T2DM) rates are also rising, and in older adults in particular [2]. Some of the most dramatic changes that occur with aging are loss of skeletal muscle mass, strength, and function [3], also known in their extreme as sarcopenia. Sarcopenia is associated with worsening disability (both ADL and IADL) [4], a higher risk of falls, fractures, and mortality [5, 6].

Sarcopenia is more common in older adults with T2DM and has recently been identified as a diabetes related complication [7, 8]. In a meta-analysis of 63 studies, including 39,581 individuals, 31% of individuals with T2DM had sarcopenia (as compared to 16.2% in controls) [9]. Coexistence of sarcopenia and T2DM in older adults is associated with poor clinical outcomes including frailty and physical disability [8, 10], sleep disorders [11], albuminuria [12], diabetic foot disease [13] and cardiovascular disease [14]. Moreover, the accurate and timely diagnosis of sarcopenia in patients with T2DM is crucial since it may lead to specific therapeutic interventions, including nutritional therapy, strength exercise training, adequate assessment of other complications and adjustment of medication to prevent hypoglycemia [7].

Diagnosis of sarcopenia necessitates both low muscle strength and documentation of low muscle quantity or quality. While magnetic resonance imaging (MRI) and computed tomography (CT) are considered the most accurate tools to assess muscle quantity, these methods are costly and often unavailable. Dual-energy X-ray absorptiometry (DXA) is a reference method for the assessment of body composition in the research field due to its fast acquisition time, low radiation exposure and relatively low cost. The method measures the attenuation of low-emission X-rays as they pass through body tissues (high attenuation through bone and low attenuation through fat) [15] in the whole body and in standard regional body composition measurements (trunk, arms, legs, android and gynoid regions) [16]. DXA estimates for body composition have been extensively compared to other body composition assessment techniques such as hydrostatic weighing, CT and MRI [17–20], and are increasingly used as a reference tool for newer body composition techniques. The main weaknesses of DXA are its limited availability, its inability to measure very tall or obese individuals and the effect of body thickness on muscle mass measurements [21].

Measurement of body composition by electrical conduction instruments (Body Impedance Analyze; BIA) is a widely used method for assessing body composition. The method measures the electrical properties of body tissue and evaluates body composition parameters that include total-body water (TBW), lean body mass (LBM or fat free mass, FFM) and fat mass (FM). BIA is a non-invasive, affordable, portable, and reliable method of body composition evaluation. The basic principle of the BIA is that the transit time of a low-voltage electric current through the body depends on the characteristics of the body composition [22]. However, this methodology has potential limitations resulting from the chemical composition of FFM (i.e., water, proteins, glycogen, and minerals) due to marked intrapersonal variability as a result of FFM changes that occur with growth, maturation, aging, and disease [23].

Older adults with T2DM may have significant alterations in body fluids due to coexisting diabetes related complications (such as heart failure [24]), therapeutic agents [such as thiazolidinediones [25], insulin [26] and sodium-glucose cotransporter-2 (SGLT-2) inhibitors [27] among others] and direct effects of hyperglycemia [28], which may potentially affect the validity of body composition assessment using BIA. Previous studies assessed the validity of a commonly used direct segmental multi-frequency bioelectrical impendence analysis (DSMF-BIA) tool (InBody analyzer) in the general middle-aged adult population [29] as well as in obese middle-aged women [30]. However, to the best of our knowledge, no studies reported the validity of DSMF-BIA in older adults with T2DM. A recent review paper published by Sbrignadello et al. [31] concluded that even though in many papers sarcopenia measurements were evaluated among patients with diabetes using BIA method, there is a necessity to test its validity in such a population. Therefore, the aim of this study was to test the agreement between the DSMF-BIA and DXA in older adults with T2DM at baseline and during treatment with commonly used therapeutic interventions, which may affect body composition and body fluids (including diet, exercise and empagliflozin).

## Methods/design

### Trial design and study sample

The CEV-65 study [32] took place at the institute of Endocrinology, Metabolism, and Hypertension (IEMH), Tel-Aviv Sourasky Medical Center between May 2018 and February 2021. The study was approved by the Tel-Aviv Sourasky Medical Center Institutional Review Board. The present study is a post hoc analysis conducted in a population sampled from this study.

Over 150 older adults with T2DM were screened according to the eligibility criteria described in [32]. The main inclusion criteria were T2DM (in accordance with American Diabetes Association guidelines [33]), age ≥65 years, HbA1C ≥ 6.5% and ≤8% and low physical activity level (based on self-report of ≥2 days a week of any leisure/exercise activity using a questionnaire adapted from the national Israeli health and nutrition survey [MABAT] for the older people, which included a list of different physical activities where subjects had to report the duration, intensity and frequency of each activity) [32]. Main exclusion criteria included treatment with a sodium-glucose transport protein 2 inhibitor (SGLT-2), recent use of steroid agents (<6 months, replacement therapy was allowed); uncorrected hypothyroidism; estimated glomerular filtration rate (eGFR) <45 cc/ml; advanced neuropathy; active participance in resistance training and/ or nutritional therapy; recent dietary change (<1 month) and/or actively participating in a weight-loss program [32].

Details of the full study design of the CEV-65 study are available in [32] (PRS: NCT03560375). Briefly, after screening and collection of baseline measurements, subjects were randomly allocated to a 10-week intervention period in one of three groups: 1. circuit resistance training (CRT) consisting of two training sessions followed by three independent home sessions/week; 2. “vegeterranean diet” (V-Med)—an ad-libitum plant-based Mediterranean diet (limited consumption of eggs, dairy and fish, avoidance of red meat and poultry); 3. empagliflozin 10 mg once daily. The final study population included 100 older adults with T2DM (60 women). Eighty-four participants (49 women) had a body composition assessment both by DXA; (Lunar Prodigy, GE Healthcare, Madison, WI, USA) and DSMF-BIA (InBody 770 body composition analyzer, Cerritos, CA, USA) at baseline. After 10 weeks of intervention 7 (4 women), 10 (5 women) and 11 (8 women) participants had both measurements of DXA and DSMF-BIA in the CRT, diet or empagliflozin intervention groups, respectively.

### Ascertainment of body composition

All measurements were done in the morning (before 10 am). Participants were asked to fast for at least 8 h prior to measurements and to avoid any intentional physical activity at the morning of the examination.

DSMF-BIA: Measurements were obtained using a InBody 770 body composition analyzer (Cerritos, CA, USA) in the standing position. Thirty bioimpedance measurements were based on six different frequencies (1 kH, 5 kHz, 50 kHz, 250 kHz, 500 kHz, 1000 kHz) at each body segment (right arm, left arm, trunk, right leg, and left leg).

DXA: DXA scan was performed using a Lunar Prodigy DXA scanner (GE Healthcare, Madison, WI, USA). Regional lean mass (kg), total-body fat (kg), and total-body fat percentage (%) were calculated using enCORE 2010 software platform version 13.31.016. Scan modes (thick, standard, or thin) were automatically set by the software. Scan times lasted approximately 5–10 min. All scan analyses were performed according to the manufacturer’s guidelines by the same technician (blinded to allocation) using standard analysis modules. In addition to total-body composition, regional estimates were made for the arms, legs, and trunk. This was accomplished by manually adjusting cut positions for each region of interest (ROI).

Appendicular skeletal mass index (ASMI) was calculated as the sum of the lean mass in the arms and legs divided by height squared (kg/m^2^).

### Other relevant measurements and definitions

A detailed list of the outcomes assessed in the CEV-65 trial can be found in ref. [32]. Height and waist circumference (measured around the umbilicus) were measured twice, and the average was then calculated, according to a standardized protocol. Weight was assessed with minimal clothing by the Inbody analyzer suitable for weighing up to 270 kg. Glycemic control was determined by fasting plasma glucose and HbA1c. Sarcopenia was defined by low appendicular lean/skeletal mass index according to cut-off values suggested by the European Working Group on Sarcopenia in Older People (EWGSOP) (<7.0 kg/m^2^ for men and <5.5 kg/m^2^ for women) [34, 35].

### Statistical methods

Statistical analyses were performed using SPSS (version 20.0, IBM, Chicago, IL, USA). Descriptive statistics of the variables are reported as means ± standard deviations (SD).

We used two methods to evaluate the degree of agreement between the DXA indices and DSMF-BIA: (1) Bland–Altman plots; (2) the Intraclass Correlation Coefficient (ICC). We used a Bland–Altman plot with regression analysis, to show the differences between the indices, vs. their mean. The agreement limit was measured at a confidence interval of 95% and was used to assess a possible relationship of gaps between the measurements and the true value (i.e., proportional bias). The existence of a proportional bias (defined here as a *p*-value < 0.05) indicates that the methods do not agree evenly on the range of the measurements (i.e., the limits of the agreement will depend on the actual measurement) [36]. ICC calculation was conducted using “Two-way mixed absolute agreement with average measures” methods. The coefficients of ICC (*r*) are estimated as follows: *r* > 0.9 is considered a very high degree of consent; 0.75 < *r* < 0.9 a high degree of consent; 0.5 < r < 0.75 a medium degree of consent; *r* < 0.5 a low degree of consent [37]. Sex stratified means were analyzed and the effect modification of sex for the agreement between the DSMF-BIA and the DXA was tested. *p*-values < 0.05 were defined statistically significant.

## Results

### General characteristics of the participants

**Fig. 1:**
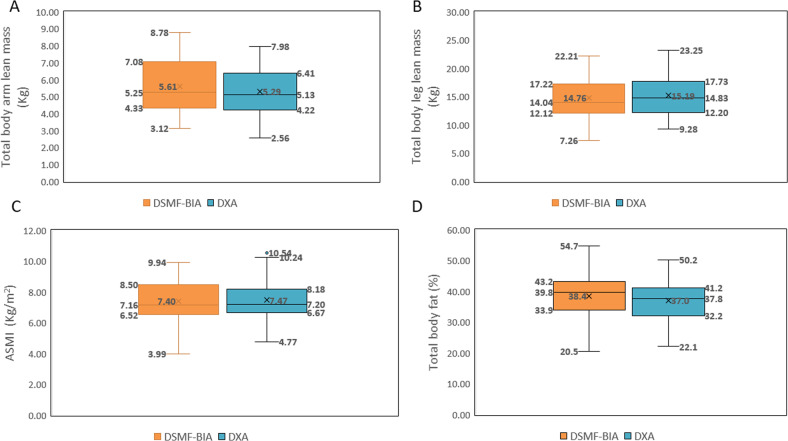
Box plots distribution of DSMF-BIA and DXA lean, ASMI and % body fat values. Box plots showing the mean values (represented by X), median values (inner line within the box), interquartile range (IQR) (Q1–Q3) values and lines indicating variability outside the upper and lower quartiles (minimum = Q1 – 1.5*IQR; maximum = Q3 + 1.5*IQR) values of: **A** arms lean body mass; **B** legs lean body mass; **C** ASMI; **D** % fat. DSMF-BIA distribution is represented by the left box in each sub-figure; DXA distribution is represented by the right box in each sub-figure. ASMI appendicular skeletal mass index, DSMF-BIA direct segmental multi-frequency bioelectrical impendence analysis, DXA dual-energy X-ray absorptiometry.

Baseline subject characteristics are detailed in Table 1. Overall mean age was 71.4 ± 5.3 years, mean BMI was 30.0 ± 5.6 kg /m^2^, mean HbA1C was 7.66% ± 1.32 and mean diabetes duration was 15.5 ± 10.0 years. Seven subjects (8%; 4 men and 3 women) were classified as sarcopenic according to DXA using muscle mass thresholds [34, 35]. Box plots distribution of DSMF-BIA and DXA lean, ASMI and % body fat values are shown in Fig. 1 indicating slightly overestimation and higher variability of arm LBM and % total-body fat using DSMF-BIA.

**Table 1:** Data of the study participants for whom baseline measurements were performed on both DSMF-BIA and DXA.

Variable^a^	Overall (*n* = 84)	Men (*n* = 35)	Women (*n* = 49)
Age (years)	71.4 ± 5.3	70.7 ± 4.3	72.0 ± 5.5
Height) meters)	1.65 ± 0.09	1.73 ± 0.07	1.59 ± 0.05
Weight (kg)	82 ± 18	93 ± 16	74 ± 14
Body mass index (BMI) kg/m^2^	30.0 ± 5.6	31.3 ± 5.6	29.0 ± 5.5
Waist circumference (cm)	105.4 ± 13.9	112.4 ± 13.1	100.3 ± 12.3
Body fat (%)—by DXA	36.98 ± 6.5	33.8 ± 6	39.3 ± 5.9
Sarcopenia according to muscle mass thresholds^b^ (*N*)—by DXA	7	4	3
Glycated hemoglobin (HbA1c; %)	7.66 ± 1.32	7.72 ± 1.45	7.61 ± 1.24
Glucose (mg/dl)	147.5 ± 40. 9	144.0 ± 41.0	150.3 ± 41.1
Diabetes duration (years)	15.5 ± 10.0	11.6 ± 9.1	18.3 ± 9.8

*DXA* dual-energy X-ray absorptiometry, *DSMF-BIA* direct segmental multi-frequency bioelectrical impedance analysis device.

^a^Variables are presented as mean ± standard deviation.

^b^Based on appendicular lean mass index <7.0 kg/m^2^ for men and <5.5 kg/m^2^ for women as defined in refs. [34, 35].

### Body composition—DSMF-BIA vs. DXA

The results of DSMF-BIA and DXA are shown in Table 2. Mean levels of lean mass segments significantly differed between the methods with a maximal difference of *–*1.29 kg (limit of agreement between −4.80 to 2.21 kg) in lean trunk mass (underestimation of DSMF-BIA). Appendicular mass segments were significantly different (−430 and 320 g differences in legs and arms lean mass, respectively). The ASMI (kg/m^2^) did not significantly differ. Fat differences between the methods were significantly different with a maximal arm fat mass difference overestimated by 2.32 kg (limit of agreement between −1.15 to 5.79 kg; *p* < 0.0001) using DSMF-BIA. Sex was not found to be an effect modifier (sex stratified mean levels of both lean and fat segments can be found in the supplementary file; Table S1).

**Table 2:** Mean levels and agreement of body composition measures assessed by DSMF-BIA and DXA among older adults with T2DM.

Variable^a^	Means comparison^a^			Bland–Altman				Intraclass correlation coefficient^b^	
	DXA	DSMF-BIA	*p*-value^c^	Bias^d^	Limit of agreement^e^	Function^f^	*p*-value^c^	ICC	*p*-value^c^
Lean mass components									
Arms lean body mass (kg)	5.29 ± 1.37	5.61 ± 1.60	*p* < 0.0001	0.31	−1.04 to 1.67	*Y* = 0.16*X* – 0.58	0.001	0.933	*p* < 0.0001
Legs lean body mass (kg)	15.19 ± 3.52	14.76 ± 3.62	*p* < 0.0001	−0.43	−2.36 to 1.49	*Y* = 0.03*X* – 0.86	0.353	0.957	*p* < 0.0001
Trunk lean body mass (kg)	24.21 ± 5.01	22.91 ± 4.86	*p* < 0.0001	−1.29	−4.80 to 2.21	*Y* = −0.3*X* – 0.53	0.425	0.950	*p* < 0.0001
ASMI (kg/m^2^)^g^	7.47 ± 1.29	7.43 ± 1.28	0.84	−0.068	−1 to 0.86	*Y* = −0.022*X* – 0.231	0.595	0.965	*p* < 0.0001
Fat mass components									
% Fat	36.98 ± 6.46	38.44 ± 6.71	*p* < 0.0001	1.46	−3.24 to 6.15	*Y* = 0.04*X* + 0.04	0.356	0.954	*p* < 0.0001
Arms fat mass (kg)	3.21 ± 1.58	5.55 ± 2.72	*p* < 0.0001	2.32	−1.15 to 5.79	*Y* = 0.59*X* – 0.23	<0.0001	0.615	*p* < 0.0001
Legs fat mass (kg)	8.92 ± 3.47	8.54 ± 2.55	0.043	−0.38	−3.79 to 3.04	*Y* = –0.33*X* + 2.45	<0.0001	0.907	*p* < 0.0001
Trunk fat mass (kg)	17.48 ± 5.57	16.30 ± 4.88	*p* < 0.0001	−1.17	−4.83 to 2.49	*Y* = –0.13*X* + 1.09d	0.001	0.955	*p* < 0.0001

*ASMI* appendicula skeletal mass index, *DXA* dual-energy X-ray absorptiometry, *DSMF-BIA* direct segmental multi-frequency bioelectrical impedance analysis device, *ICC* intraclass correlation coefficient, *T2DM* type 2 diabetes.

^a^Variables are presented as mean ± standard deviation and differences between DSMF-BIA method and DXA in each body composition parameter were examined by paired sample *t*-test.

^b^ICC > 0.9 = excellent; ICC 0.9–0.75 = good; ICC 0.75–0.5 = moderate; ICC < 0.5 = poor.

^c^For means comparison and Bland–Altman tests a *p* < 0.05 represents a significant difference or a significant bias, whereas for intraclass correlation coefficient a *p* < 0.05 represents a significant correlation.

^d^Mean bias is considered the mean difference for each measurement between DSMF-BIA and DXA.

^e^Limit of agreement = mean bias ± 1.96 SD.

^f^A regression analysis using ordinary least squares regression.

^g^Appendicular lean mass index was calculated as the sum of the lean mass in the arms and legs divided by height squared (kg/m^2^).

#### Agreement between DSMF-BIA and DXA

**Fig. 2:**
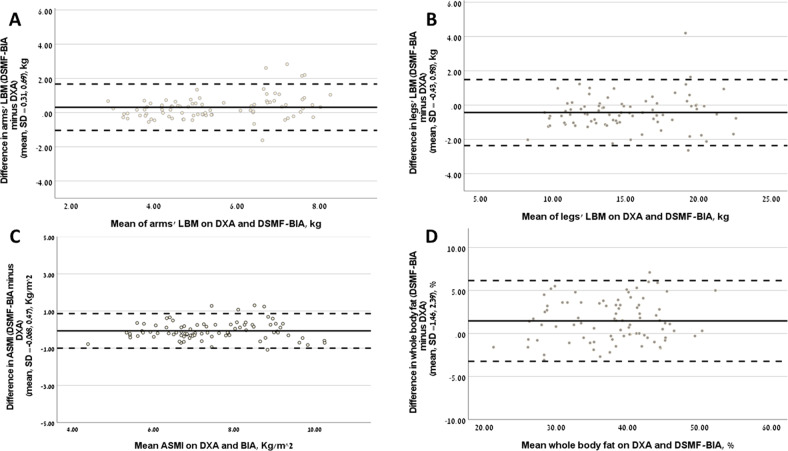
Bland–Altman analysis of the degree of agreement between DSMF-BIA and DXA. Bland–Altman plots presenting the difference between DSMF-BIA and DXA vs. mean value of **A** arms lean body mass; **B** legs lean body mass; **C** ASMI; **D** % fat. The solid line represents the mean bias and the broken line the ±1.96 SD. Mean bias is considered the mean difference (of all individuals) between DSMF-BIA and DXA. ASMI appendicular skeletal mass index, DSMF-BIA direct segmental multi-frequency bioelectrical impendence analysis, DXA dual-energy X-ray absorptiometry, LBM lean body mass.

Examination of the agreement between the methods according to specific parameters of body composition are presented in Table 2, Fig. 2 and by sex in Table S1 and Fig. S1.

- Agreement using intraclass correlation coefficient (ICC)
  Comparison of the two methods in the total cohort, using ICCs showed excellent correlations (ICC > 0.9) with a *p*-value <0.05 in all measurements (lean and fat) aside from fat arm (ICC = 0.615) (Table 2). When analyzed by sex (Table S1), there were good and excellent agreements between the methods in measuring regional lean mass in limbs (arms and legs) and trunk among men and women (ICC ≥ 0.872, all *p* < 0.001), except for moderate-grade agreement in the parameter of arms lean mass in men (ICC = 0.706, *p* < 0.001). For both sexes, excellent correlations were found for body fat composition (% fat and FM in different regions) (ICC ≥ 0.873, *p* < 0.001 for all), excluding FM in the arms (ICC (men) = 0.483; ICC (women) = 0.749; *p* < 0.001 for both) (Table S1).
- Agreement using Bland–Altman method
  The degree of agreement was further tested using the Bland–Altman method (Table 2, Table S1, Fig. 2, Fig. S1). Comparing the agreement between the methods on arms LBM showed a mean difference of 0.31 ± 0.69 kg, which was significant (limits of agreement, −1.04 to 1.67 kg, *p* = 0.001) (Fig. 2A and Table 2). For lean mass in the legs and trunk, as well as for ASMI, there was an agreement between the methods (Fig. 2B, C and Table 2), which was observed also when analyzed by sex (Table S1 and Fig. S1).
  There was no agreement between the DSMF-BIA and the DXA methods for all fat measures except total fat percentage (Table 2 and Fig. 2D). Total fat percentage bias was 1.46 ± 2.39 % (*p* = 0.356; 95% limits of agreement −3.24 to 6.15 %) with most measurements obtained by DSMF-BIA overestimating results obtained by DXA. Agreement for total fat percentage remained for sex when analyzed separately (Table S1 and Fig. S1D).
- Agreement between the methods following therapeutic interventions
  Following 10 weeks of empagliflozin, CRT or a V-Med diet the trends for lean mass remained with arm LBM overestimated, legs LBM and ASMI underestimated, and fat percentage overestimated by the DSMF-BIA in all interventions (Table 3 and Fig. S2). Despite having profound effects on body composition, the different interventions did not seem to affect the high agreement between DXA and DSMF-BIA.

**Table 3:** Agreement of body composition measures assessed by DSMF-BIA and DXA among older adults with T2DM—after 10 weeks of V-MED, CRT, or empagliflozin.

Method	Bland–Altman Plot				ICC^a^	
	Bias^b^	Limit of agreement^c^	Function^d^	*p*-value^e^		*p*-value^e^
V-Med (*N* = 10)						
LBM arm	0.4	−1.47 to 2.28	*Y* = 0.17*X* – 0.59	0.468	0.884	*p* < 0.05
LBM leg	−0.33	−2.25 to 1.59	*Y* = −0.01*X* – 0.14	0.892	0.985	*p* < 0.001
ASMI	0.003	−1.22 to 1.22	*Y* = −0.021*X* + 0.161	0.907	0.946	*p* < 0.001
Fat %	1.51	−2.17 to 5.19	*Y* = −0.22*X* + 9.74	0.129	0.933	*p* < 0.001
CRT (*N* = 7)						
LBM arm	0.35	−0.45 to 1.16	*Y* = −0.03*X* + 0.52	0.786	0.979	*p* < 0.001
LBM leg	−0.67	−2.56 to 1.22	*Y* = −0.12*X* + 1.25	0.287	0.977	*p* < 0.001
ASMI	−0.12	−0.82 to 0.59	*Y* = −0.142*X* + 0.989	0.106	0.989	*p* < 0.001
Fat %	0.86	−4.76 to 6.48	*Y* = −0.03*X* + 2.05	0.902	0.935	*p* < 0.001
Empagliflozin (*N* = 11)						
LBM arm	0.05	−0.97 to 1.08	*Y* = 0.14*X* – 0.614	0.274	0.967	*p* < 0.001
LBM leg	−0.53	−1.44 to 0.37	*Y* = 0.09*X* – 1.74	0.088	0.984	*p* < 0.001
ASMI	−0.20	−0.81 to 0.41	*Y* = 0.143*X* – 1.204	0.089	0.976	*p* < 0.001
Fat%	1.55	−2.05 to 5.16	*Y* = −0.012*X* + 2	0.890	0.975	*p* < 0.001

*ASMI* appendicular skeletal muscle index, *CRT* circuit resistance training, *DXA* dual-energy X-ray absorptiometry, *DSMF-BIA* direct segmental multi-frequency bioelectrical impedance analysis device, *ICC* intraclass correlation coefficient, *LBM* lean body mass, *V-MED* vegeterranean diet, *T2DM* type 2 diabetes.

^a^ICC > 0.9 = excellent; ICC 0.9–0.75 = good; ICC 0.75–0.5 = moderate; ICC < 0.5 = poor.

^b^Mean bias is considered the mean difference for each measurement between DSMF-BIA and DXA.

^c^Limit of agreement = mean bias ± 1.96 SD.

^d^A regression analysis using ordinary least squares regression.

^e^For Bland–Altman tests a *p* < 0.05 represents a significant difference or a significant bias, whereas for intraclass correlation coefficient a *p* < 0.05 represents a significant correlation.

### The validity of DSMF-BIA device as compared to the DXA device in diagnosing sarcopenia

Lastly, we compared specificity/sensitivity and positive/negative predictive values of DSMF-BIA compared to DXA for diagnosing sarcopenia (Table S2). When compared to DXA, DSMF-BIA had high specificity (93%) and high negative predictive value (97%).

## Discussion

Our results clearly show that assessing body composition and diagnosing sarcopenia using the DSMF-BIA method is comparable to the DXA scan in older adults with T2DM. This was not affected by a 10-week intervention period with treatment modalities often used in the treatment of T2DM. The degree of agreement between the methods was overall better when comparing parameters of LBM than parameters of FM. For diagnosing sarcopenia in the clinical setting, DSMF-BIA had high specificity and high negative predictive value, suggesting it may be a useful screening tool for this condition.

Discrepancies between DXA and DSMF-BIA have been previously noted in different populations. In a previous study in healthy individuals undergoing a 4-week low calorie diet, DSMF-BIA slightly over estimated fat free mass and underestimated FM and % fat—although there was no statistically significant difference between these methods [38]. An underestimation of FM and % fat and overestimation of FFM results were also seen in other studies comparing DXA to different DSMF-BIA devices (InBody230, InBody720, and InBody770) in healthy men and women [39–41]. In contrast, in older subjects with T2DM, BF and % fat were slightly overestimated while correlation was high. This discrepancy highlights the importance of validation of DSMF-BIA in different populations and under different physiologic interventions. Given the increasing rates of diabetes in general and in older adults in particular [2] and the wide use of BIA methods in studies testing diabetes and sarcopenia outcomes [31], it is not surprising that there was a call for validation studies testing the accuracy of BIA for this population [31].

Using DXA as a method to diagnose sarcopenia has several inherent limitations. DXA scan is less accessible and more expensive than DSMF-BIA. Very tall and very obese individuals cannot be adequately measured in a standard DXA machine and body thickness and hydration status (e.g., water retention, heart kidney, or liver failure) can affect muscle mass measurement [42]. Moreover, to ensure the accuracy of any method for assessing muscle mass, standardization is needed. Calibration of materials and equations used to derive lean mass should be standardized across manufacturers. It is important to standardize the local regions of interest, such as trunk, arms, legs, which are different across manufacturers. Finally, defining a reference population in the same way as has been achieved for the use of DXA in diagnosing osteoporosis should be considered [43].

The strengths of this manuscript include its relatively large population, its prospective nature and focus on a relatively poorly studied population of older adults with diabetes and low physical activity level. Moreover, we present the lack of a significant effect of commonly used interventions for the treatment of T2DM on the validity of DSMF-BIA. Its weaknesses include the limited sample size in the prospective phase, the relatively short period of follow up and the fact that the study was not specifically designed to validate MF-BIA vs. DXA. Also, the validity tested in the current paper is limited to relatively young older adults with T2DM who have limited rate of complications (with low levels of sarcopenia). In that sense the significance of the findings presented here are important for early detecting lower muscle mass for early prevention, but with the cost of limited external validity.

In conclusion, the DSMF-BIA as compared to DXA is a reliable screening technique for sarcopenia in older patients with T2DM. Accurate and accessible diagnosis of sarcopenia is crucial in older subjects with diabetes and directly affects clinical decisions and treatment [44–46]. The routine use of DSMF-BIA as a screening tool for sarcopenia in clinics treating older patients with diabetes should be considered.

## Supplementary information

Supplemental material

## Data Availability

The datasets used and/or analyzed during the current study are available from the corresponding author on reasonable request.
