# Supplementary material for: Validation of a multi-frequency bioelectrical impedance analysis device for the assessment of body composition in older adults with type 2 diabetes
Source: Nutr Diabetes. 2022 Oct 20;12:45. doi: 10.1038/s41387-022-00223-1 (PMC9584890; doi:10.1038/s41387-022-00223-1)
Supplement: Supplementary file 1 — Supplemental material [file 41387_2022_223_MOESM1_ESM.docx]

**Validation of a multi-frequency bioelectrical impedance analysis device for the assessment of body composition in older adults with type 2 diabetes**

**Supplementary file**

**Table S1: Mean levels and agreement of body composition measures assessed by DSMF-BIA and DXA among older adults with T2DM – stratified by gender**

| *Variable^1^* | *Means comparison^1^* | | | *Bland- Altman* | | | | *Intra Class Correlation Coefficient^5^* | |
| --- | --- | --- | --- | --- | --- | --- | --- | --- | --- |
|  | ***DXA*** | ***DSMF-BIA*** | ***p value^6^*** | ***Bias^2^*** | ***Limit  of agree-ment^3^*** | ***Function^4^*** | ***p value^6^*** | **ICC** | ***p value^6^*** |
| *Lean mass components* | | | | | | | | | |
| *Arms lean body mass (Kg)* | | | | | | | | | |
| *Men* | 6.54± 0.83 | 7.07±1.1 | 0.001 | 0.52 | -1.15 to 2.19 | Y= 0.33X-1.73 | 0.045 | 0.706 | p<0.001 |
| *Women* | 4.4±0.90 | 4.56±0.96 | 0.032 | 0.16 | -0.83 to 1.15 | Y= 0.07X-0.15 | 0.402 | 0.915 | p<0.001 |
| *Legs lean body mass (Kg)* | | | | | | | | | |
| *Men* | 18.31±2.59 | 17.96±2.67 | 0.106 | -0.35 | -2.78 to 2.09 | Y= 0.03X-0.9 | 0.721 | 0.938 | p<0.001 |
| *Women* | 12.96±2.11 | 12.47± 2.18 | P < 0.0001 | -0.49 | -1.96 to 0.98 | Y= 0.03X-0.9 | 0.538 | 0.956 | p<0.001 |
| *Trunk lean body mass (Kg)* | | | | | | | | | |
| *Men* | 28.41±3.42 | 27.4±3.3 | 0.001 | -1.05 | -4.48 to 2.37 | Y= -0.05X+0.2 | 0.636 | 0.905 | p<0.001 |
| *Women* | 21.2±3.62 | 19.7±2.9 | P < 0.0001 | -1.46 | -5.02 to 2.10 | Y= -0.23X+3.24 | 0.004 | 0.872 | p<0.001 |
| *ASMI (Kg/m^2^)^7^* | | | | | | | | | |
| *Men* | 8.35±1.05 | 8.37±0.96 | 0.934 |  |  |  |  | 0.910 | p<0.001 |
| *Women* | 6.84±1.06 | 6.77±1.05 | 0.743 |  |  |  |  | 0.965 | p<0.001 |
| *Fat mass components* | | | | | | | | | |
| *% Fat* |  |  |  |  |  |  |  |  |  |
| *Men* | 33.8 ±6 | 35.3±6.5 | 0.001 | 1.5 | -3.33 to 6.33 | Y= 0.09X-1.56 | 0.205 | 0.946 | p<0.001 |
| *Women* | 39.3 ± 5.9 | 40.7±5.9 | P < 0.0001 | 1.43 | -3.22 to 6.07 | Y= 0.02X+0.76 | 0.782 | 0.944 | p<0.001 |
| *Arms fat mass (Kg)* | | | | | | | | | |
| *Men* | 2.95±1.28 | 5.95±3.05 | P < 0.0001 | 2.96 | -1.36 to 7.27 | Y= 0.89X-0.98 | <0.0001 | 0.483 | p<0.001 |
| *Women* | 3.39±1.75 | 5.27±2.44 | ***P < 0.0001*** | 1.88 | -0.55 to 4.31 | Y= 0.35X-0.35 | <0.0001 | 0.749 | p<0.001 |
| *Legs fat mass (Kg)* | | | | |  | | | | |
| *Men* | 8.52±3.5 | 8.57±2.61 | 0.845 | 0.09 | -2.42 to 2.59 | Y= -0.34X+2.28 | <0.0001 | 0.956 | p<0.001 |
| *Women* | 9.21±3.44 | 8.51±2.52 | 0.016 | -0.7 | -4.52 to 3.12 | Y= -0.34X+2.28 | <0.0001 | 0.873 | p<0.001 |
| *Trunk fat mass (Kg)* | | | | | | | | | |
| *Men* | 19.83± 6.21 | 17.6±5.4 | < 0.0001 | -2.22 | -6.17 to 1.73 | Y= -0.14X+0.42 | 0.017 | 0.936 | p<0.001 |
| *Women* | 15.81±4.4 | 15.4±4.3 | 0.028 | -0.44 | -3.11 to 2.23 | Y= -0.03X-0.02 | 0.526 | 0.973 | p<0.001 |

^1^Variables are presented as mean ± standard deviation and differences between DSMF-BIA method and DXA in each body composition parameter were examined by paired sample T test;

^2^ Mean bias is considered the mean difference for each measurement between DSMF-BIA and DXA;

^3^ mean bias±1.96 SD;

^4^ a regression analysis using ordinary least squares regression.

^5^ ICC > 0.9 = excellent; ICC 0.9-0.75 = good; ICC 0.75-0.5 = moderate; ICC < 0.5 = poor.

^6^ For means comparison and Bland-Altman tests a p <0.05 represents a significant difference or a significant bias, whereas for intra class correlation coefficient a p<0.05 represents a significant correlation;

^7^ Appendicular lean mass index was calculated as the sum of the lean mass in the arms and legs divided by height squared (Kg/m^2^).

Abbreviations: ASMI, appendicula skeletal mass index; DXA, dual-energy X-ray absorptiometry; DSMF-BIA, direct segmental multi-frequency bioelectrical impedance analysis device; ICC= Intra Class Correlation Coefficient; T2DM, Type 2 diabetes.

**Table S2: Validity and predictive values for DSMF-BIA compared to the reference method (DXA) among older adults with T2DM^1^**

|  | ***Overall*** | ***Men*** | ***Women*** |
| --- | --- | --- | --- |
| ***Sensitivity (%)^2^*** | 71% (5/7) | 75% (3/4) | 66% (2/3) |
| ***Specificity (%)^3^*** | 93% (72/77) | 93% (29/31) | 93% (43/46) |
| ***Positive predictive value (%)*** | 50% (5/10) | 60% (3/5) | 40% (2/5) |
| ***Negative predictive value (%)*** | 97% (72/74) | 97% (29/30) | 98% (43/44) |

^1^ A diagnosis of sarcopenia (defined here as a ‘positive’ results) is based on appendicular lean mass index <7.0 kg/m^2^ for men and <5.5 kg/m^2^ for women as defined in (1,2).

^2^ The proportion of true positives according to DSMF-BIA out of the true positives classified by DXA

^3^ The proportion of true negatives according to DSMF-BIA out of the true positives classified by DXA

^4^ The proportion of true positives according to DSMF-BIA out of the positives classified by DSMF-BIA (true positives by DSMF-BIA + false positives by DSMF-BIA)

^5^ The proportion of true negatives according to DSMF-BIA out of the negatives classified by DSMF-BIA (true negatives by DSMF-BIA + false negatives by DSMF-BIA)

**Fig S1 Bland Altman analysis of the degree of agreement between DSMF-BIA and DXA in men and women**


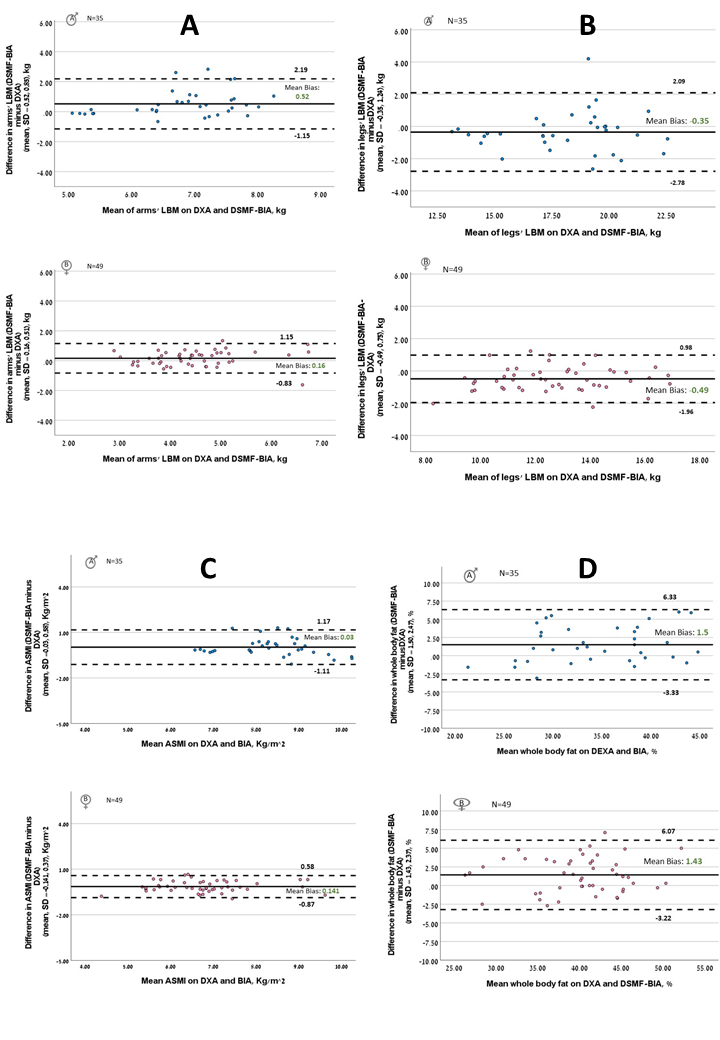


Fig. S1. Bland–Altman plots presenting the difference between DSMF-BIA and DXA vs. mean value for: A. arms lean body mass; B. legs lean body mass; C. ASMI; D. % fat. Among men and women. The solid line represents the mean bias and the broken line the ±1.96 SD. Mean bias is considered the mean difference (of all individuals; men or women) between DSMF-BIA and DXA.

**Fig S2** *Bland Altman analysis of the degree of agreement between DSMF-BIA and DXA after 10 weeks of V-MED, CRT or empagliflozin*


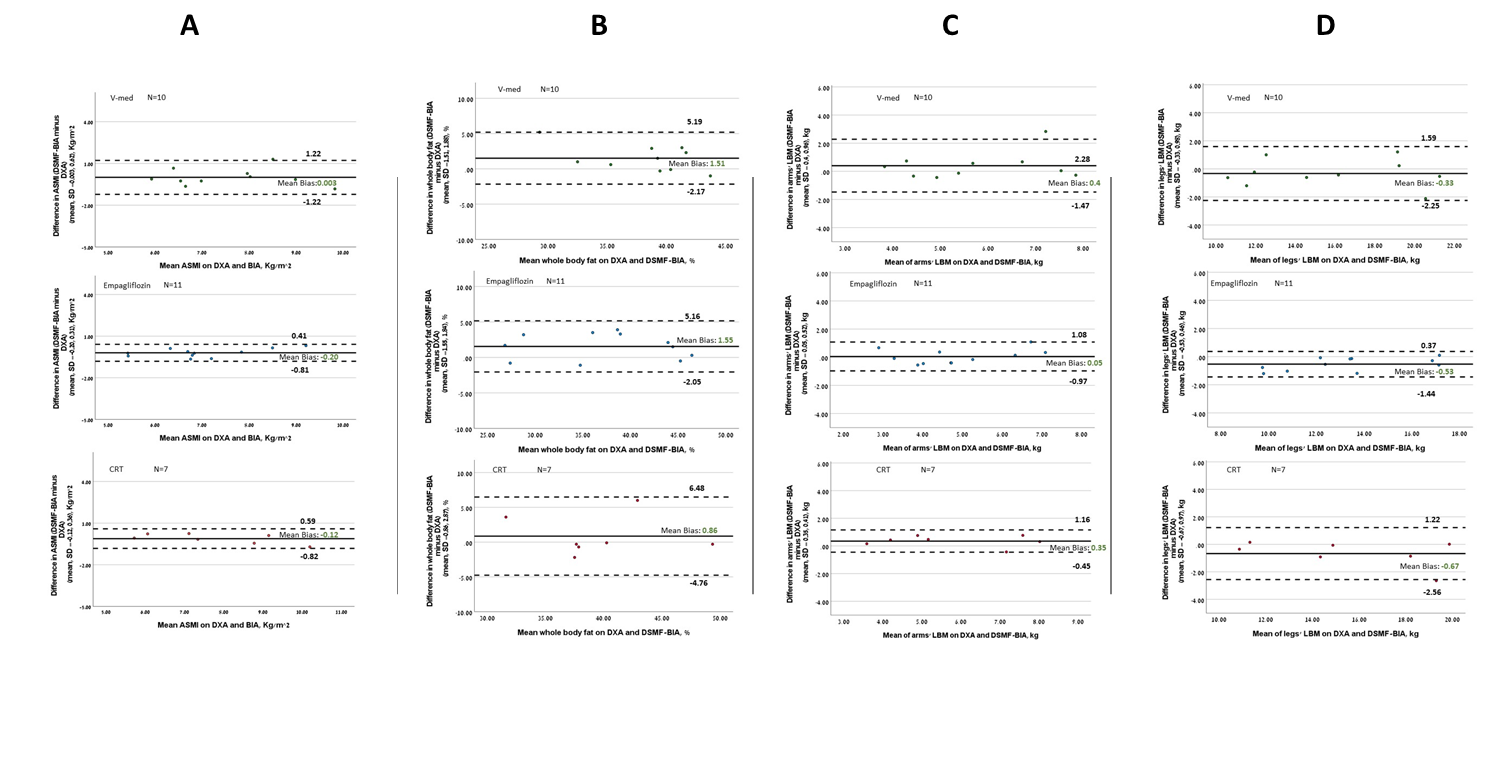


**Supplementary Figure 2. Bland Altman analysis of the degree of agreement between DSMF-BIA and DXA** **after 10 weeks of V-MED, empagliflozin or CRT.** Bland–Altman plots presenting the difference between DSMF-BIA and DEXA vs. mean value of A. arm lean body mass; B. legs lean body mass; C. ASMI; D. fat percentage measured on DEXA and DSMF-BIA (each sub-figure is stratified by the three interventions - V-MED, empagliflozin and CRT). The solid line represents the mean bias and the broken line the ±1.96 SD. Mean bias is considered the mean difference (of all individuals; men and women) between DSMF-BIA and DXA. Abbreviations: ASMI, Appendicular skeletal mass index; CRT, Circuit Resistance Training; LBM- lean body mass; V-MED, vegeterranean diet.

# References

1. Cruz-Jentoft AJ, Bahat G, Bauer J, Boirie Y, Bruyère O, Cederholm T, et al. Sarcopenia: revised European consensus on definition and diagnosis. Age Ageing. 2019 Jan 1;48(1):16–31.

2. Gould H, Brennan SL, Kotowicz MA, Nicholson GC, Pasco JA. Total and Appendicular Lean Mass Reference Ranges for Australian Men and Women: The Geelong Osteoporosis Study. Calcif Tissue Int. 2014 Apr 1;94(4):363–72.
